# Supplementary material for: Intrinsic and non-cell autonomous roles for a neurodevelopmental syndrome-linked transcription factor
Source: bioRxiv. 2025 Dec 25:2025.12.23.696256. Preprint. [Version 1] doi: 10.64898/2025.12.23.696256 (PMC12776094; doi:10.64898/2025.12.23.696256)
Supplement: Supplement 4 [file media-4.pdf]

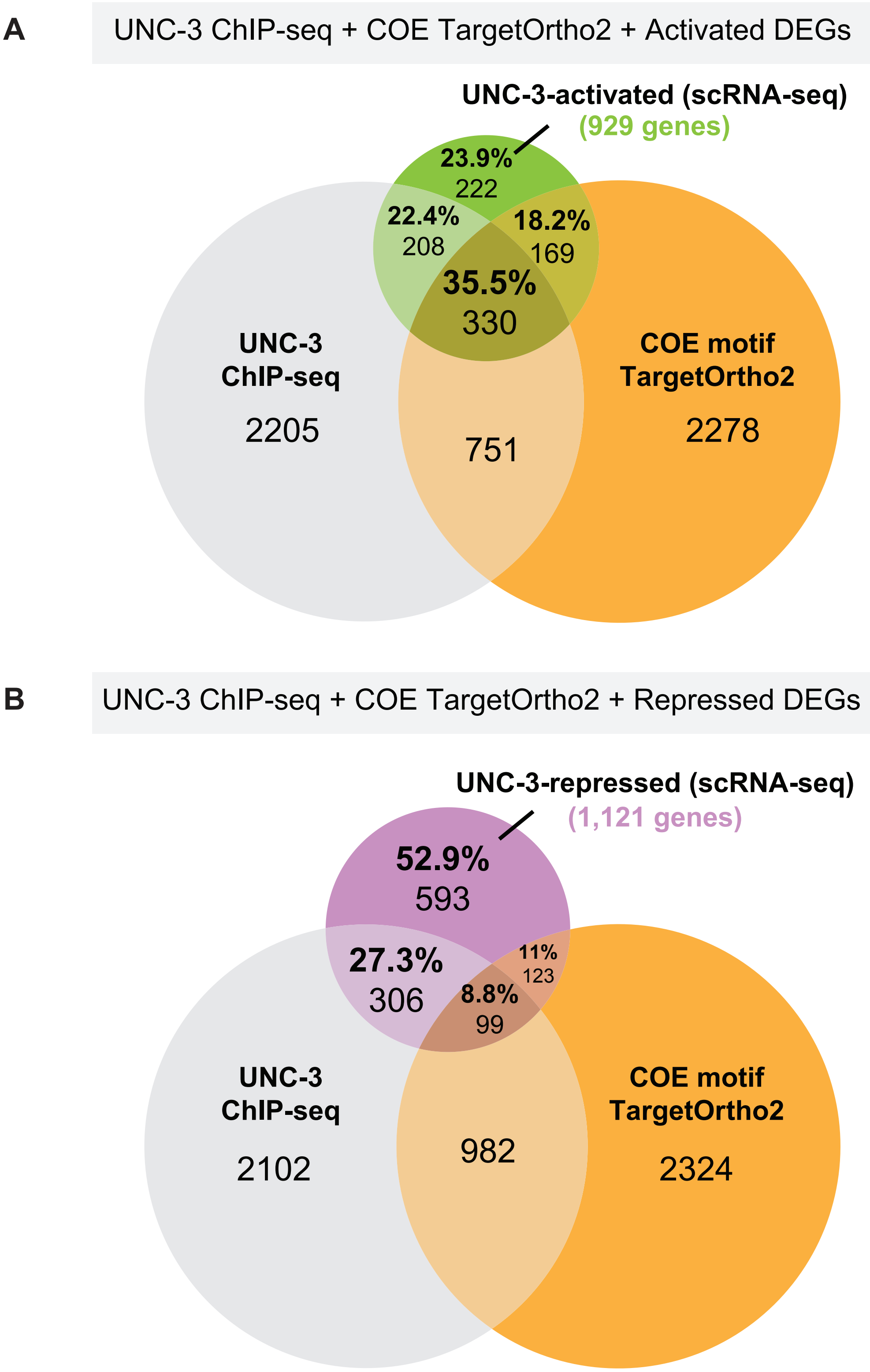

Figure S4. Integration of genomic and transcriptomic datasets supports COE-independent repression by UNC-3. (A-B) Venn diagrams depicting the proportion of activated (A) or repressed (B) DEGs shared among UNC-3 ChIP-seq targets (Li et al., 2020), COE TargetOrtho2 predicted targets (Rumley et al., 2025), or both.
