# Supplementary figures and images for: Intrinsic and non-cell autonomous roles for a neurodevelopmental syndrome-linked transcription factor
Source: bioRxiv. 2025 Dec 25:2025.12.23.696256. Preprint. [Version 1] doi: 10.64898/2025.12.23.696256 (PMC12776094; doi:10.64898/2025.12.23.696256)

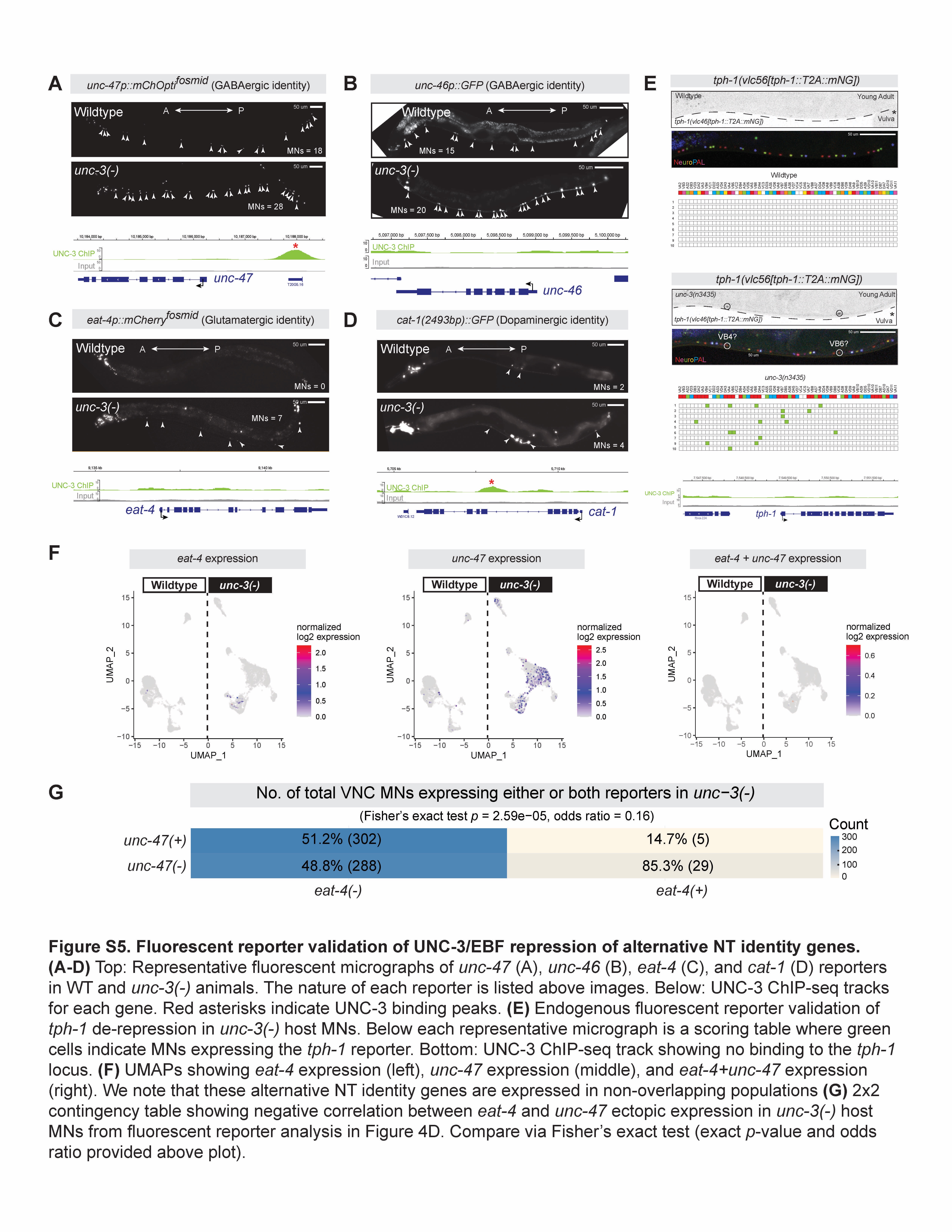

Supplement: Supplement 5 [file media-5.jpg]
