# Supplementary material for: Intrinsic and non-cell autonomous roles for a neurodevelopmental syndrome-linked transcription factor
Source: bioRxiv. 2025 Dec 25:2025.12.23.696256. Preprint. [Version 1] doi: 10.64898/2025.12.23.696256 (PMC12776094; doi:10.64898/2025.12.23.696256)
Supplement: Supplement 6 [file media-6.pdf]

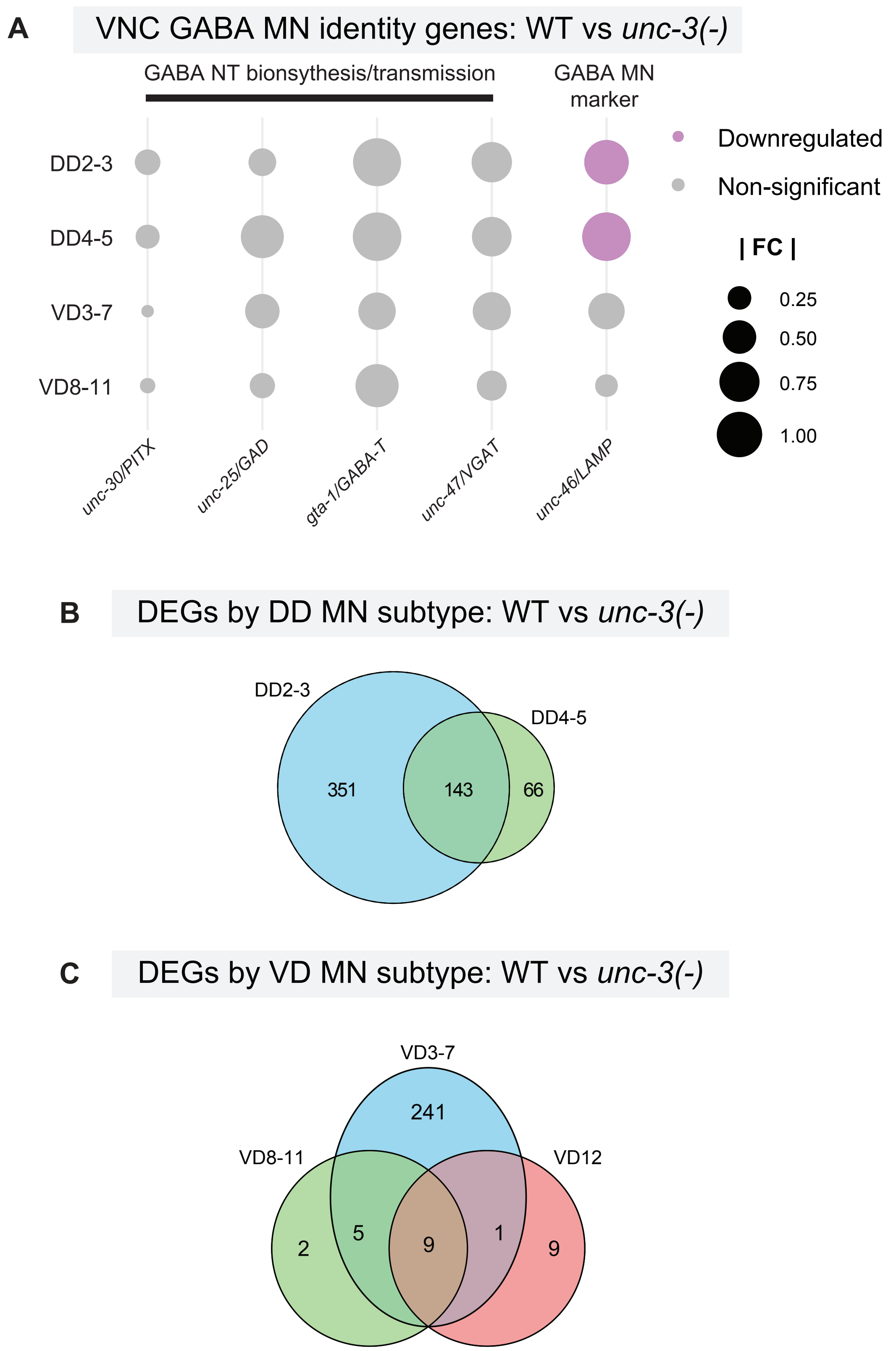

Figure S6. GABA NT identity is intact in downstream MNs of *unc-3(-)* mutants. (A) Dot plot depicting no dysregulation of known GABA MN NT identity genes in GABA MN subclasses of *unc-3(-)* animals. However, we note that the GABA MN identity marker *unc-46/LAMP* is downregulated in the absence of *unc-3* (validated in Fig. S8). **(B-C)** Venn diagrams depicting the number of unique and shared DEGs across DD (B) and VD (C) GABA MN subclasses.
