# Supplementary material for: Intrinsic and non-cell autonomous roles for a neurodevelopmental syndrome-linked transcription factor
Source: bioRxiv. 2025 Dec 25:2025.12.23.696256. Preprint. [Version 1] doi: 10.64898/2025.12.23.696256 (PMC12776094; doi:10.64898/2025.12.23.696256)
Supplement: Supplement 12 [file media-12.pdf]

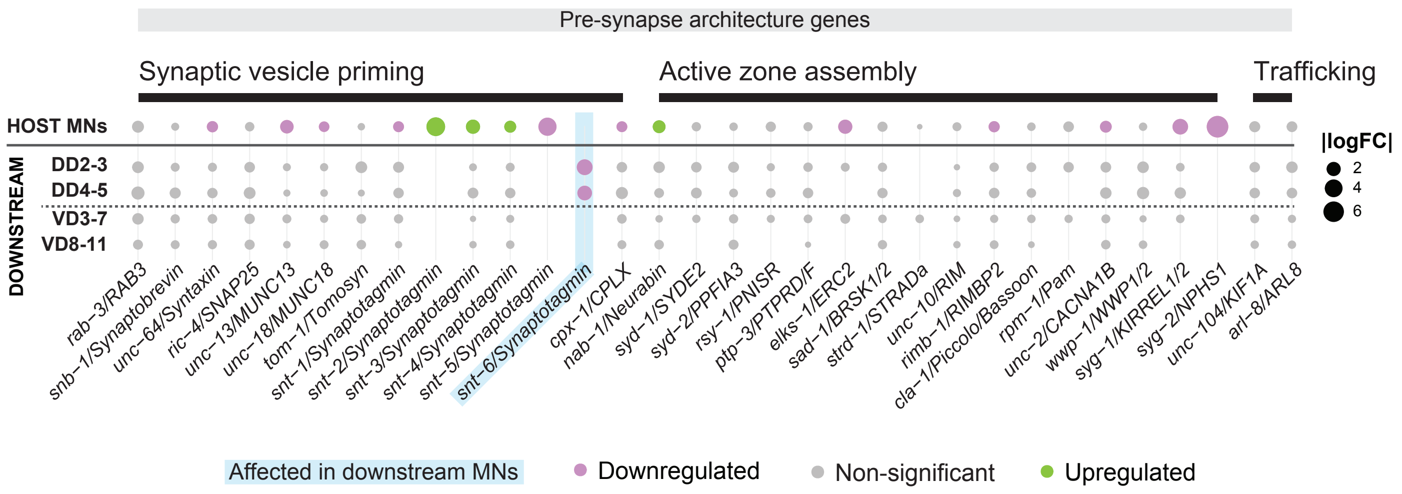


**Figure S12. Expression of presynaptic genes in host and downstream (GABA) MNs in the absence of *unc-3*.** Dot plot depicting DEGs involved in synaptic vesicle priming (left) or active zone assembly (right) in host or downstream MNs.
