# Supplementary material for: Intrinsic and non-cell autonomous roles for a neurodevelopmental syndrome-linked transcription factor
Source: bioRxiv. 2025 Dec 25:2025.12.23.696256. Preprint. [Version 1] doi: 10.64898/2025.12.23.696256 (PMC12776094; doi:10.64898/2025.12.23.696256)
Supplement: Supplement 20 [file media-20.pdf]

Homer *de novo* Motif Results

Downregulated Genes

Total target sequences = 650
Total background sequences = 41701
* - possible false positive

Rank	Motif	P-value	log P-pvalue	% of Targets	% of Background	STD(Bg STD)	Best Match
1	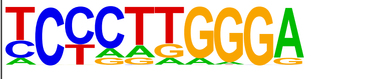	1e-40	-9.390e+01	8.92%	0.77%	18.2bp (23.5bp)	POL013.1_MED-1/Jaspar(0.502)
2	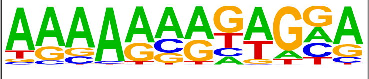	1e-21	-4.950e+01	30.92%	15.73%	20.7bp (28.0bp)	blmp-1/MA0537.1/Jaspar(0.639)
3	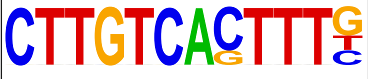	1e-13	-3.155e+01	0.92%	0.00%	19.9bp (9.8bp)	blmp-1/MA0537.1/Jaspar(0.727)
4 *	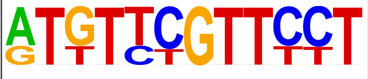	1e-11	-2.560e+01	0.77%	0.00%	11.5bp (11.2bp)	MF0009.1_TRP(MYB)_class/Jaspar(0.638)
5 *	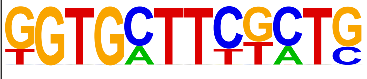	1e-11	-2.560e+01	0.77%	0.00%	15.8bp (7.7bp)	POL008.1_DCE_S_I/Jaspar(0.564)
6 *	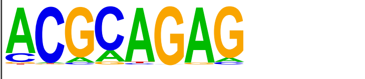	1e-10	-2.309e+01	14.00%	6.83%	19.7bp (28.4bp)	eor-1/MA0543.1/Jaspar(0.659)
7 *	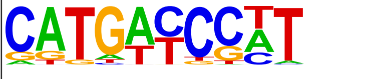	1e-9	-2.163e+01	2.77%	0.41%	20.5bp (22.3bp)	MF0004.1_Nuclear_Receptor_class/Jaspar(0.662)
8 *	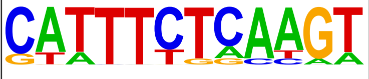	1e-9	-2.123e+01	2.46%	0.32%	17.7bp (23.1bp)	ceh-22/MA0264.1/Jaspar(0.627)
9 *	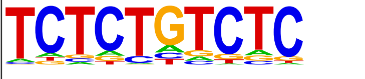	1e-9	-2.097e+01	16.77%	9.18%	18.0bp (37.6bp)	eor-1/MA0543.1/Jaspar(0.803)
10 *	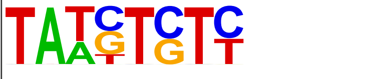	1e-8	-2.070e+01	20.92%	12.47%	18.9bp (26.0bp)	unc-62/MA0918.1/Jaspar(0.576)
11 *	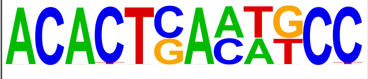	1e-8	-2.013e+01	0.77%	0.01%	11.8bp (12.6bp)	MF0011.1_HMG_class/Jaspar(0.509)
12 *	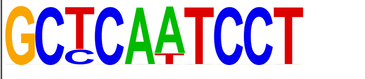	1e-8	-1.982e+01	0.62%	0.00%	19.7bp (4.2bp)	unc-30/MA1443.1/Jaspar(0.610)
13 *	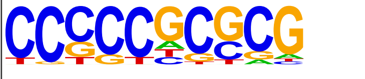	1e-8	-1.916e+01	4.77%	1.38%	16.1bp (27.4bp)	dpy-27/MA0540.1/Jaspar(0.578)
14 *	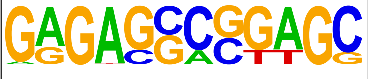	1e-8	-1.876e+01	2.62%	0.43%	16.9bp (25.3bp)	POL013.1_MED-1/Jaspar(0.594)
15 *	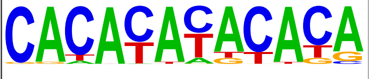	1e-7	-1.814e+01	3.69%	0.91%	18.2bp (27.6bp)	daf-12/MA0538.1/Jaspar(0.755)
16 *	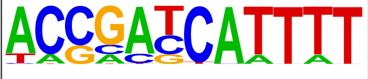	1e-7	-1.813e+01	1.54%	0.12%	16.9bp (26.0bp)	POL002.1_INR/Jaspar(0.541)
17 *	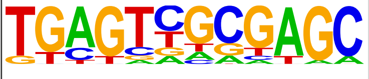	1e-7	-1.786e+01	2.46%	0.40%	21.1bp (20.8bp)	POL010.1_DCE_S_III/Jaspar(0.602)
18 *	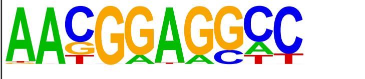	1e-7	-1.760e+01	0.77%	0.01%	23.3bp (15.8bp)	POL013.1_MED-1/Jaspar(0.635)

						(32.9bp)	
20 *	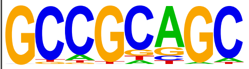	1e-6	-1.468e+01	5.08%	1.87%	16.7bp (23.6bp)	POL013.1_MED-1/Jaspar(0.640)
21 *	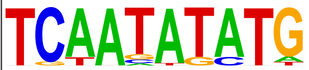	1e-6	-1.466e+01	1.38%	0.14%	18.9bp (21.1bp)	MF0008.1_MADS_class/Jaspar(0.670)
22 *	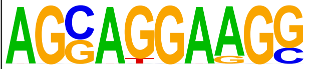	1e-5	-1.358e+01	1.23%	0.12%	16.3bp (21.3bp)	POL008.1_DCE_S_I/Jaspar(0.661)
23 *	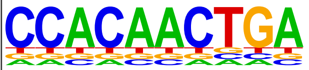	1e-5	-1.221e+01	0.46%	0.01%	20.5bp (14.2bp)	hlh-1/MA0545.1/Jaspar(0.632)
24 *	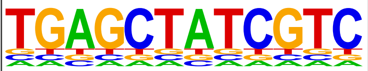	1e-5	-1.221e+01	0.46%	0.01%	5.4bp (12.2bp)	nhr-6/MA1451.1/Jaspar(0.518)
25 *	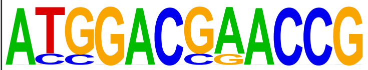	1e-5	-1.221e+01	0.46%	0.01%	14.9bp (5.3bp)	MF0009.1_TRP(MYB)_class/Jaspar(0.557)
26 *	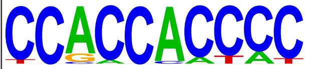	1e-5	-1.195e+01	1.69%	0.30%	15.0bp (28.1bp)	POL003.1_GC-box/Jaspar(0.617)
27 *	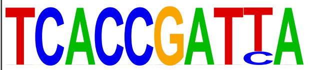	1e-4	-1.015e+01	0.46%	0.01%	17.9bp (13.6bp)	ceh-28/MA1445.1/Jaspar(0.606)
28 *	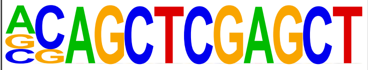	1e-3	-9.017e+00	0.31%	0.00%	16.7bp (0.0bp)	POL010.1_DCE_S_III/Jaspar(0.544)
29 *	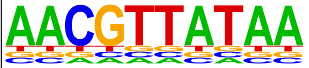	1e-3	-7.641e+00	0.31%	0.01%	22.0bp (10.8bp)	zip-8/MA1704.1/Jaspar(0.622)
30 *	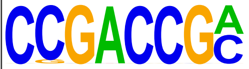	1e-2	-6.118e+00	0.92%	0.20%	13.9bp (23.9bp)	POL011.1_XCPE1/Jaspar(0.535)

Homer Known Motif Enrichment Results

Downregulated Genes

Total Target Sequences = 650, Total Background Sequences = 41501

Rank	Motif	Name	P-value	log P-pvalue	q-value (Benjamini)	# Target Sequences with Motif	% of Targets Sequences with Motif	# Background Sequences with Motif	% of Background Sequences with Motif
1	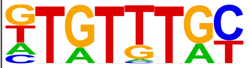	PHA-4(Forkhead)/cElegans-Embryos-PHA4-ChIP-Seq(modEncode)/Homer	1e-3	-7.413e+00	0.0066	105.0	16.15%	4897.1	11.80%
2	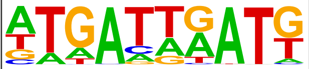	LIN-39(Homeobox)/cElegans.L3-LIN39-ChIP-Seq(modEncode)/Homer	1e-2	-4.978e+00	0.0379	58.0	8.92%	2646.8	6.38%
