# Supplementary material for: Intrinsic and non-cell autonomous roles for a neurodevelopmental syndrome-linked transcription factor
Source: bioRxiv. 2025 Dec 25:2025.12.23.696256. Preprint. [Version 1] doi: 10.64898/2025.12.23.696256 (PMC12776094; doi:10.64898/2025.12.23.696256)
Supplement: Supplement 21 [file media-21.pdf]

Homer *de novo* Motif Results

Upregulated Genes

Total target sequences = 412
Total background sequences = 38500
* - possible false positive

Rank	Motif	P-value	log P-pvalue	% of Targets	% of Background	STD(Bg STD)	Best Match/Details
1	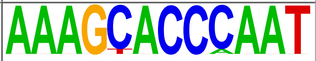	1e-20	-4.761e+01	2.67%	0.02%	17.6bp (13.8bp)	ceh-10::ttx-3/MA0263.1/Jaspar(0.622)
2	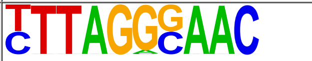	1e-19	-4.376e+01	2.91%	0.04%	16.9bp (23.8bp)	MF0006.1_bZIP_cEBP-like_subclass/Jaspar(0.668)
3	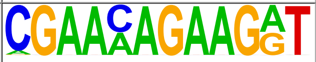	1e-18	-4.371e+01	2.67%	0.02%	17.6bp (14.2bp)	POL008.1_DCE_S_I/Jaspar(0.583)
4	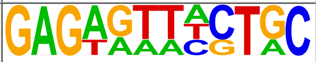	1e-18	-4.267e+01	2.67%	0.03%	21.0bp (25.1bp)	daf-12/MA0538.1/Jaspar(0.558)
5	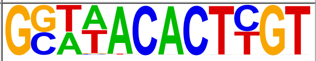	1e-16	-3.694e+01	1.94%	0.01%	16.3bp (17.7bp)	daf-16/MA1446.1/Jaspar(0.580)
6	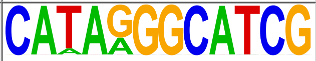	1e-15	-3.640e+01	1.70%	0.01%	13.5bp (21.5bp)	PL0012.1_hlh-2::hlh-8/Jaspar(0.534)
7	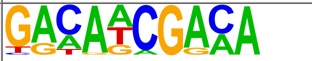	1e-14	-3.324e+01	5.83%	0.68%	18.3bp (24.6bp)	blmp-1/MA0537.1/Jaspar(0.669)
8	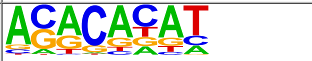	1e-14	-3.305e+01	33.01%	17.15%	19.7bp (27.2bp)	sma-4/MA0925.1/Jaspar(0.662)
9	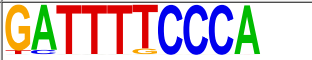	1e-13	-3.058e+01	2.91%	0.11%	22.4bp (21.3bp)	MF0003.1_REL_class/Jaspar(0.642)
10	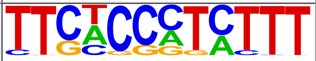	1e-12	-2.978e+01	4.13%	0.33%	21.2bp (21.5bp)	eor-1/MA0543.1/Jaspar(0.579)
11	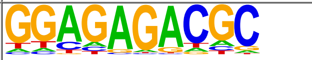	1e-12	-2.970e+01	12.14%	3.60%	20.2bp (29.4bp)	eor-1/MA0543.1/Jaspar(0.657)
12 *	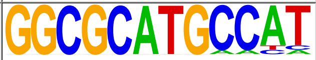	1e-11	-2.632e+01	1.46%	0.01%	10.4bp (17.2bp)	PL0018.1_hlh-25/Jaspar(0.593)
13 *	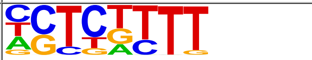	1e-9	-2.287e+01	31.55%	18.45%	20.0bp (29.6bp)	blmp-1/MA0537.1/Jaspar(0.636)
14 *	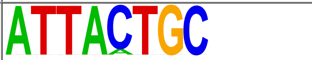	1e-9	-2.117e+01	2.91%	0.24%	21.6bp (23.7bp)	MF0010.1_Homeobox_class/Jaspar(0.685)
15 *	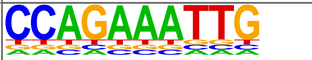	1e-8	-2.031e+01	1.46%	0.03%	20.5bp (14.6bp)	sma-4/MA0925.1/Jaspar(0.692)
16 *	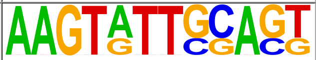	1e-8	-1.846e+01	1.21%	0.02%	15.1bp (11.5bp)	MF0010.1_Homeobox_class/Jaspar(0.596)
17 *	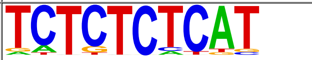	1e-7	-1.719e+01	6.07%	1.63%	24.8bp (34.9bp)	blmp-1/MA0537.1/Jaspar(0.581)
18 *	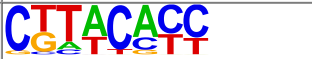	1e-7	-1.690e+01	8.98%	3.26%	19.5bp (25.2bp)	SD0001.1_at_AC_acceptor/Jaspar(0.671)
19 *	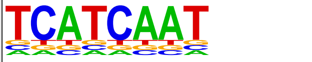	1e-7	-1.685e+01	2.91%	0.36%	20.4bp (19.5bp)	LIN-39(Homeobox)/cElegans.L3-LIN39-ChIP-Seq(modEncode)/Homer(0.726)
20 *	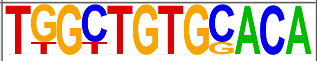	1e-6	-1.580e+01	0.73%	0.00%	8.7bp (0.0bp)	POL009.1_DCE_S_II/Jaspar(0.603)
21 *	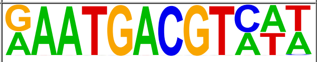	1e-6	-1.410e+01	1.21%	0.04%	20.2bp (20.1bp)	atf-7/MA1438.1/Jaspar(0.865)
22 *	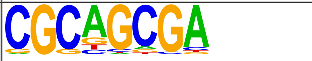	1e-5	-1.322e+01	7.04%	2.60%	17.9bp (28.3bp)	dpy-27/MA0540.1/Jaspar(0.656)
23 *	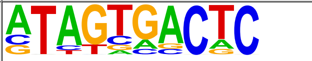	1e-5	-1.318e+01	2.67%	0.42%	17.9bp (22.8bp)	fos-1/MA1448.1/Jaspar(0.684)
24 *	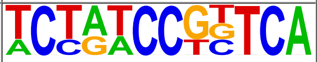	1e-5	-1.234e+01	0.97%	0.03%	18.1bp (12.5bp)	POL004.1_CCAAT-box/Jaspar(0.571)
25 *	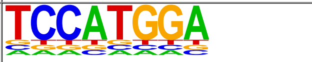	1e-4	-1.136e+01	1.21%	0.07%	20.2bp (13.7bp)	ceh-48/MA0921.1/Jaspar(0.495)

Homer Known Motif Enrichment Results

Upregulated Genes

Total Target Sequences = 412, Total Background Sequences = 43733

Rank	Motif	Name	P-value	log P-pvalue	q-value (Benjamini)	# Target Sequences with Motif	% of Targets Sequences with Motif	# Background Sequences with Motif	% of Background Sequences with Motif
1	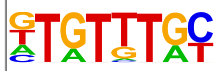	PHA-4(Forkhead)/cElegans-Embryos-PHA4-ChIP-Seq(modEncode)/Homer	1e-3	-8.003e+00	0.0037	79.0	19.17%	5733.4	13.11%
2	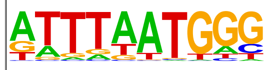	EGL-5(Homeobox)/cElegans-L3-EGL5-ChIP-Seq(modEncode)/Homer	1e-3	-7.941e+00	0.0037	50.0	12.14%	3208.9	7.33%
3	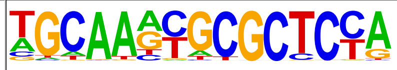	EFL-1(E2F)/cElegans-L1-EFL1-ChIP-Seq(modEncode)/Homer	1e-3	-7.816e+00	0.0037	11.0	2.67%	334.6	0.76%
4	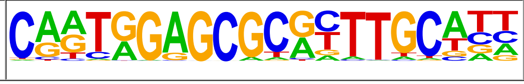	LIN-15B(Zf)/cElegans-L3-LIN15B-ChIP-Seq(modEncode)/Homer	1e-3	-7.811e+00	0.0037	10.0	2.43%	281.3	0.64%
