# Supplementary material for: Intrinsic and non-cell autonomous roles for a neurodevelopmental syndrome-linked transcription factor
Source: bioRxiv. 2025 Dec 25:2025.12.23.696256. Preprint. [Version 1] doi: 10.64898/2025.12.23.696256 (PMC12776094; doi:10.64898/2025.12.23.696256)
Supplement: Supplement 10 [file media-10.pdf]

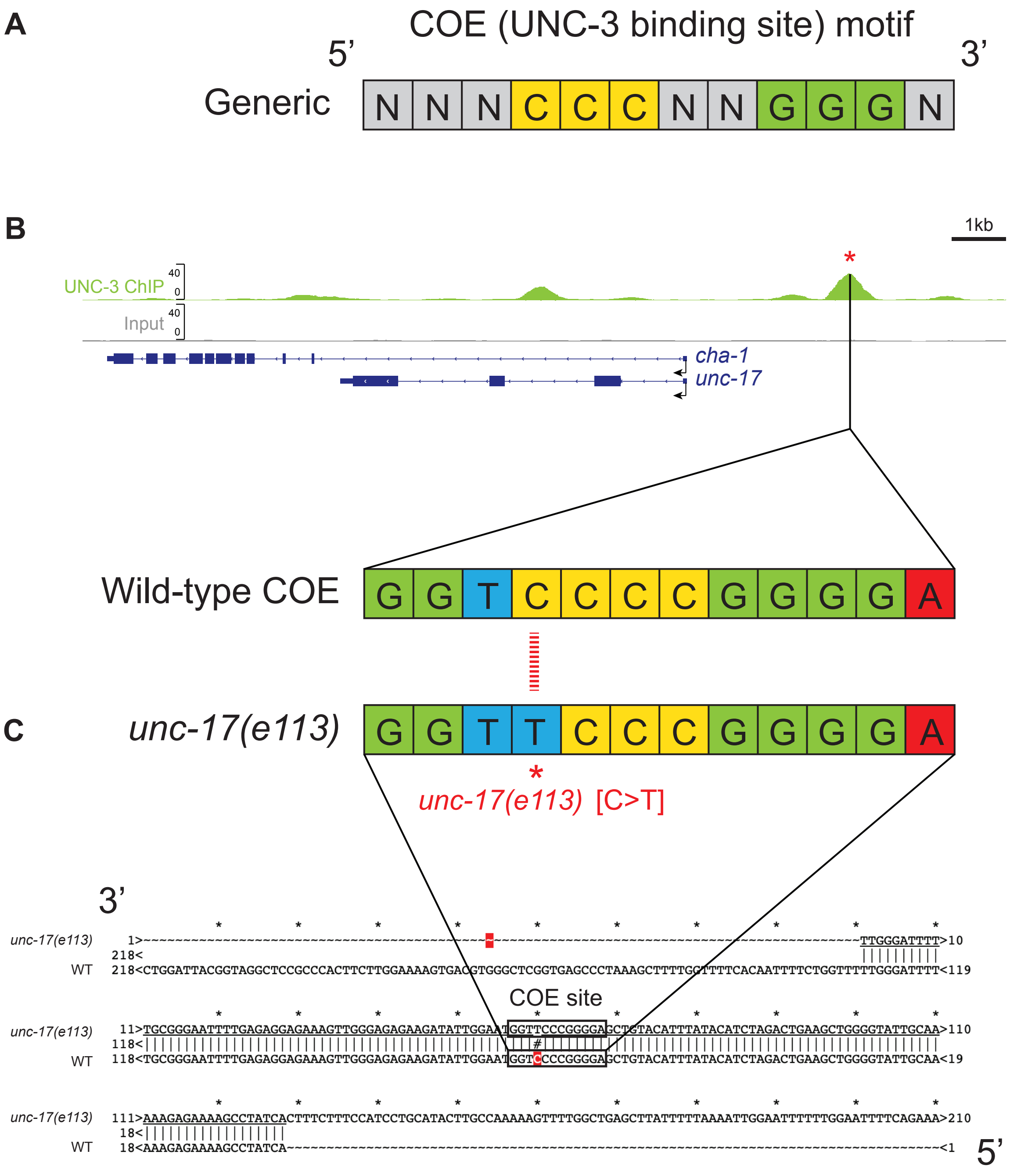

Figure S10. The *unc-17(e113)* variant targets the COE motif upstream of the *cha-1/unc-17* locus. **(A)** The sequence of UNC-3's cognate binding site for activation, the COE motif. The letter N refers to variable identities of nucleotides in that position, whereas all other letters refer to specific nucleotide identities: A = Adenine, T = Thymine, C = Cytosine, G = Guanine. **(B)** Top: UNC-3 ChIP-seq track showing binding in a single COE-containing peak ~2kb upstream of the *cha-1/unc-17* locus. Bottom: exact COE sequenced found with the UNC-3 ChIP-seq peak. (C) Sanger sequencing revealed that the *unc-17(e113)* variant is a C>T point mutation affecting a deeply conserved residue in the COE motif corresponding to the site of UNC-3 binding.
